# Promoting an active choice among physically inactive adults: a randomised web-based four-arm experiment

**DOI:** 10.1186/s12966-022-01288-y

**Published:** 2022-04-27

**Authors:** Lorraine L. Landais, Olga C. Damman, Judith G. M. Jelsma, Evert A. L. M. Verhagen, Danielle R. M. Timmermans

**Affiliations:** 1 Department of Public and Occupational Health, Amsterdam UMC location Vrije Universiteit Amsterdam, Boelelaan 1117, Amsterdam, The Netherlands; 2 Department of Public and Occupational Health, Amsterdam Movement Sciences, Amsterdam Collaboration On Health & Safety in Sports, Amsterdam UMC location Vrije Universiteit Amsterdam, Amsterdam, The Netherlands

**Keywords:** Inactive Adults, Active choice, Decision making, Web-based intervention, Intention, Physical activity, Process evaluation

## Abstract

**Background:**

Promoting active (i.e., conscious, autonomous, informed, and value-congruent) choices may improve the effectiveness of physical activity interventions. This web-based four-arm experimental study investigated the effect of promoting an active versus passive choice regarding physical activity on behavioural and psychological outcomes (e.g., physical activity intentions and behaviours, autonomy, commitment) among physically inactive adults.

**Methods:**

Dutch inactive adults were randomized into four groups: physical activity guideline only (control group G), guideline & information (GI), guideline & active choice (GA), or guideline & active choice & action planning (GA +). GA and GA + participants were stimulated to make an active choice by weighing advantages and disadvantages of physical activity, considering personal values, and identifying barriers. GA + participants additionally completed action/coping planning exercises. Passive choice groups G and GI did not receive exercises. Self-reported behavioural outcomes were assessed by a questionnaire pre-intervention (T0, *n* = 564) and at 2–4 weeks follow-up (T2, *n* = 493). Psychological outcomes were assessed post-intervention (T1, *n* = 564) and at follow-up. Regression analyses compared the outcomes of groups GI, GA and GA + with group G. We also conducted sensitivity analyses and a process evaluation.

**Results:**

Although promoting an active choice process (i.e., interventions GA and GA +) did not improve intention (T1) or physical activity (T2 versus T0), GA + participants reported higher commitment at T1 (β = 0.44;95%CI:0.04;0.84) and more frequently perceived an increase in physical activity between T0 and T2 (β = 2.61;95%CI:1.44;7.72). GA participants also made a more active choice at T1 (β = 0.16;95%CI:0.04;0.27). The GA and GA + intervention did not significantly increase the remaining outcomes. GI participants reported higher intention strength (β = 0.64;95%CI:0.15;1.12), autonomy (β = 0.50;95%CI:0.05;0.95), and commitment (β = 0.39;95%CI:0.04;0.74), and made a more active choice at T1 (β = 0.13;95%CI:0.02;0.24). Interestingly, gender and health condition modified the effect on several outcomes. The GA + intervention was somewhat more effective in women. The process evaluation showed that participants varied in how they perceived the intervention.

**Conclusions:**

There is no convincing evidence of a beneficial effect of an active versus passive choice intervention on physical activity intentions and behaviours among inactive adults. Further research should determine whether and how active choice interventions that are gender-sensitized and consider health conditions can effectively increase physical activity.

**Trial registration:**

ClinicalTrials.gov: NCT04973813. Retrospectively registered.

**Supplementary Information:**

The online version contains supplementary material available at 10.1186/s12966-022-01288-y.

## Introduction

Interventions that promote physical activity among inactive adults generally show small effects, usually diminishing over time [1]. Intervention outcomes may be improved if individuals would be supported in making a more active choice about their physical activity behaviour. Previous studies in choice architecture and behavioural economics have shown that individuals’ decisions are influenced by how choices are presented in terms of content and framing [2, 3]. In this field, the term ‘active choice’ usually refers to an explicit choice between options [4–6]. We use a broader conceptualisation based on dual-process theories of decision-making [7]. We defined ‘active choice’ as a conscious and autonomous choice in which an individual is aware that there is a choice, actively weighs the advantages and disadvantages of choice options, considers personal values, and thinks about specific personal goals, potential barriers to achieving those goals, and ways to cope with those barriers. In contrast, we use ‘passive choice’ for choices that barely involve autonomy and choice awareness and lack consideration of advantages/disadvantages, values, goals, and potential barriers.

Our definition of ‘active choice’ is in line with existing cognitive-behavioural frameworks, including Motivational interviewing (MI) [8], the Disconnected Values Model (DVM) [9], Acceptance and Commitment Therapy (ACT) [10], and Implementation Intentions [11]. MI, the DVM and ACT emphasize the necessity of clarifying individuals’ values to foster commitment to behaviours consistent with those values. According to MI and the DVM, sustainable behaviour change is more likely if individuals perceive a significant discrepancy between their health behaviour and values and conclude that behaving in a more value-congruent way will reduce this discrepancy and increase life satisfaction [8, 12]. This premise is rooted in cognitive dissonance theory, which proposes that people strive for internal psychological consistency and a reduction of cognitive dissonance [13]. Furthermore, MI and the DVM interventions encourage individuals to make concrete action plans for behavioural change. Implementation intentions are well-established examples of concrete action plans in which a specific situational context (i.e., the ‘if’ component) is linked with a goal-directed cognition or behaviour (i.e., the ‘then’ component) [14]. Previous research has demonstrated that health-promoting interventions based on MI, ACT, or implementation intentions effectively increase physical activity in adults, including inactive adults and adults with a health condition [15–20]. Interventions based on the DVM have been shown to effectively increase physical fitness in university employees [21, 22].

Promoting an active choice process may improve behavioural and psychological intervention outcomes for multiple reasons. Previous research has established that providing individuals with a choice – instead of telling them more directly what to do – increases autonomy [23, 24]. Autonomy has positively been associated with self-efficacy, intrinsic motivation, and behavioural persistence [24–26]. Secondly, as active choices involve weighing advantages and disadvantages associated with choice options, active choices promote more informed choices [27], and as such foster more autonomous choices [28]. Whether individuals can make informed choices depends on multiple factors, including the quality of information or decision support and individuals’ own information-processing motivation and skills [29]. Thirdly, choices that align with people’s values increase satisfaction and commitment, inducing greater behavioural persistence [5, 30, 31]. If individuals experience ambivalence between ‘health’ and conflicting values – including social and work-related values [32, 33] – this ambivalence can be resolved by tools that help individuals to reflect on their values [34, 35]. A final advantage of an active choice process is that spelling out personal goals – by planning when, where and how goal-directed behaviour will be performed (i.e., behaviour change technique ‘action planning’) – increases self-efficacy and the likelihood that intentions are translated into behaviour [11, 36]. A meta-analytic review showed that action planning is more effective in changing physical activity behaviour if individuals also plan to cope with barriers (i.e., ‘coping planning’) [20].

In the current web-based experimental study, we used a pre-test post-test four-arm parallel design to investigate the effect of promoting an active choice regarding physical activity on self-reported behavioural outcomes (e.g., physical activity behaviour; perceived increase in physical activity) and psychological outcomes (e.g., physical activity intention; commitment) among physically inactive adults. The outcomes were compared to a control group in which we promoted a more passive choice. The intervention components, the corresponding behaviour change techniques (BCTs) [37, 38] and the specific underlying psychological theories/models are shown in Table 1. As outlined, we expected that promoting an active choice process would result in better behavioural and psychological outcomes than promoting a passive choice process.

**Table 1:** Intervention components included in each group

**Intervention component**	**Corresponding BCT** [37, 38]	**Specific theory/model**	**Group G** **(Guideline only)**	**Group GI** **(Guideline + Information)**	**Group GA** **(Guideline + Active choice)**	**Group GA + ** **(Guideline + Active choice + Action and coping planning)**
National physical activity guideline	Instruction on how to perform a behaviour		X	X	X	X
Examples of advantages and disadvantages of increasing PA	Pros and cons; Information about health consequences; Information about emotional consequences			X	X	X
Examples of barriers to PA	Barrier identification			X	X	X
Exercise 1: Describe and prioritize advantages and disadvantages of current PA and of increasing PA	Pros and cons	Decisional balance [39]			X	X
Exercise 2: (A) Indicate the importance of several values, including ‘health’; (B) indicate the time, effort and energy spent on those values; (C) compare ‘importance of health’ with ‘time, effort and energy spent on health’; (D) indicate extent to which several values affect PA	Valued self-identity; Discrepancy between current behaviour and goal	Disconnected Values Model [9]; Cognitive dissonance theory [13]			X	X
Examples of strategies to increase PA	Instruction on how to perform a behaviour					X
Exercise 3: Create personal PA action plan	Action planning; Goal setting	Action planning [40]				X^a^
Exercise 4: Indicate personal barriers	Barrier identification				X	X
Exercise 5: Make plans to cope with personal barriers (implementation intentions)	Problem solving	Implementation intentions; coping planning [11]				X^a^

*BCT* Behaviour change technique, *PA* Physical activity

^a^Only participants who intended to become more physically active completed this intervention component

## Methods

### Design and setting

**Fig. 1:**
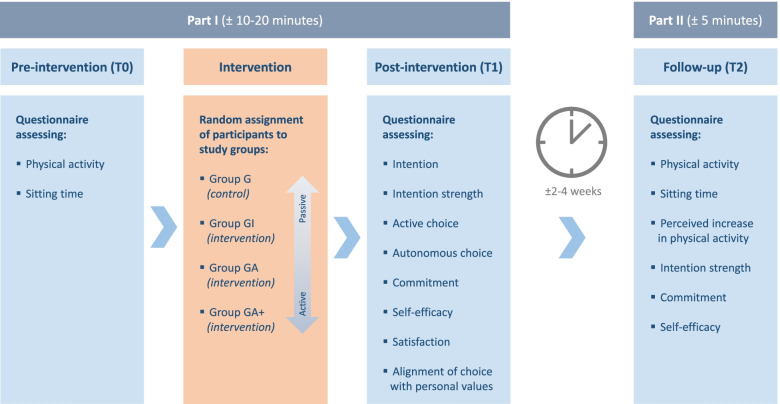
Study design

This web-based randomized controlled trial employed a pre-test, post-test four-arm parallel design. The four arms – G, GI, GA and GA + – are described in the Intervention and Control section. At baseline (T0), a questionnaire assessed behavioural outcomes. This pre-intervention questionnaire was immediately followed by the intervention and post-intervention questionnaire (T1), which assessed psychological outcomes. Approximately 2–4 weeks after this first part of the study (Part I), participants were invited to complete the second part (Part II): a follow-up questionnaire (T2) that assessed behavioural outcomes and some of the psychological outcomes. An overview of the study design is shown in Fig. 1. Although data collection was performed during the COVID-19 pandemic (September/October 2020), most public life activities – including work-outs in gyms – were allowed by the Dutch governmental measures. Our study was reviewed by Amsterdam UMC’s medical research ethics committee (2020.142).

### Participants

Participants were Dutch adult members of an online ISO certified panel of Flycatcher Internet Research (www.flycatcher.eu). Panel members signed up voluntarily and earned points for research participation, which could be exchanged for gift cards. Panel members were eligible if they reported low physical activity levels (i.e., physically active for at least 30 min on less than 5 days a week *and* engaged in less than 150 min of physical activity in total throughout an average week [41]). Pregnant women, wheelchair users and individuals not able to walk a minimum of 100 m were excluded.

### Sample size and power calculation

The study was powered to detect a 12% difference in ‘Intention to become more physically active’ (proportion ‘yes’ versus ‘no’) post-intervention (T1) between group G and group GA + using an alpha level of 0.05 and a statistical power of 80%. This meant that 182 participants were required per group. Consequently, at follow-up (T2) – assuming a drop-out rate of 33% – we would be able to detect a difference of 598 ‘metabolic equivalent of task’ (MET) minutes per week between group G and group GA + , as well as a 15% difference regarding the percentage of participants that would be ‘moderate/high active’ versus ‘low active’ [42].

### Intervention and control

The four experimental groups included a national physical activity guideline only group (control group G), guideline & information group (GI), guideline & active choice group (GA), or guideline & active choice & action planning group (GA +). Table 1 presents the intervention components included in each group. The study materials – which were all self-constructed by the authors – gradually expanded from ‘promoting a very passive choice’ (G) to ‘promoting a very active choice’ (GA +) to evaluate individual intervention components. All study materials were provided online and did not involve professional guidance. Participants were blind to the group they were assigned to.

The GA + intervention (Additional file 1) promoted an active choice about physical activity through several exercises. Exercise 1 asked to describe advantages and disadvantages of current physical activity behaviour and increasing physical activity – comparable to a ‘decisional balance sheet’ [39]. Participants were subsequently asked to select three advantages/disadvantages considered most important. Exercise 2 promoted a value-congruent choice and was based on the DVM [9] and its theoretical foundations, including cognitive dissonance theory [13]. This value-clarification exercise asked participants to indicate how important they considered health, family, friendship, performance (e.g., at work), balance (e.g., between work and private life), pleasure, and responsibility on a scale of 1 (not important) to 10 (very important). These values were selected based on our previous qualitative work [43]. Participants were subsequently asked to indicate how much time, effort and energy they spent on each value in the past year on a scale of 1 (not much) to 10 (very much). Finally, we presented their answers about ‘health’ to both questions and asked whether they perceived a discrepancy and whether they wanted to spend more time, effort, and energy on their health (yes/no). Next, we presented participants their answers for the remaining values and asked to what extent each value affected their physical activity behaviour (again on a scale of 1 to 10).

After this exercise, the Dutch physical activity guideline was presented [44], followed by examples of strategies to become more physically active. We asked participants whether they wanted to become more physically active (yes/maybe/no); those who answered ‘yes’ or ‘maybe’ were asked to make a personal action plan [11, 14], to indicate possible barriers and to make coping plans [20]. In contrast, those who answered ‘no’ were only asked to indicate possible barriers.

The GA intervention was comparable to the GA + intervention but lacked strategies to increase physical activity and action and coping planning exercises. GI participants received the physical activity guideline, supplemented with information about possible advantages and disadvantages and barriers to physical activity. The control group – group G – only received the physical activity guideline [44].

### Procedure

Before data collection, we pre-tested the GA + intervention and physical activity questionnaire among six relatively inactive adults. Based on this pre-test, we made several textual and visual adaptations.

Part I was disseminated between September 4–22, 2020. The research agency invited all panel members (*n* = 9395) by e-mail, along with information about the study topic and estimated time to complete Part I: 10–20 min. The follow-up questionnaire was not mentioned during Part I to prevent measurement reactivity [45]. After clicking the link to the study, panel members were asked five questions as an eligibility check. Eligible panel members were more fully informed about the study and data collection/storage. The research agency randomly assigned participants to one of the four groups using the ‘random sample of cases’ function for simple random sampling in SPSS.

All participants actively provided informed consent. Before the intervention, participants reported physical activity levels. Subsequently, participants completed the intervention and the questionnaire with the remaining outcome measures.

To ensure approximately equal time intervals (± 2–4 weeks) between Part I and Part II, participants completing Part I before September 10 were invited for Part II on September 24; participants completing Part I between September 10–22 were invited on September 30. All participants who at least completed T0 were invited for Part II. We closed the data collection on October 9, 2020.

### Outcomes

Our primary outcome measures were intention to become more physically active (T1) and physical activity (T2 versus T0). Table 2 shows all outcome measures, including their measurement and time of measurement. All outcome measures in this study were based on self-report.

**Table 2:** Measurement of the behavioural and psychological outcome measures

**Outcome measure**	**Item(s)**	**Scale / Response categories**	**Scale’s Cronbach’s alpha**^a^	**Time of measurement**
**Behavioural outcomes**				
Physical activity	IPAQ short form: 6 items to assess weekly time spent in: ▪ vigorous intensity activities ▪ moderate intensity activities ▪ walking	Days per week/ Hours and minutes per day/ ‘Don’t know/not sure’		T0, T2
Sitting time	IPAQ short form: 1 item to assess daily sitting time	Hours and minutes per day/ ‘Don’t know/not sure’		T0, T2
Perceived increase in physical activity	▪ *Have you changed your physical activity behaviour in the past two weeks? If yes, what have you changed?*	Yes, namely: … / No		T2
**Psychological outcomes**				
Intention	▪ *Do you plan to become more physically active?*	Yes / No		T1
Intention strength	▪ *How strong is your plan to become more physically active?*	1 (no strong plan at all)—10 (very strong plan)		T1, T2^b^
Active choice	*In making this choice [about whether or not to become more physically active], I have…* ▪ *… taken into account the advantages of my current physical activity behaviour* ▪ *… taken into account the disadvantages of my current physical activity behaviour* ▪ *… taken into account the advantages of being more physically active* ▪ *… taken into account the disadvantages of being more physically active* ▪ *… thought about the advantages and disadvantages that are most important to me* ▪ *… thought about how important my health is to me* ▪ *… thought about the difference between ‘how important my health is to me’ and ‘how much time, effort and energy I spend on it’* ▪ *… taken into account the time, effort and energy it takes to become more physically active* ▪ *… taken into account barriers to become more physically active*	1 (totally disagree)—5 (totally agree)	0.77	T1
Autonomous choice	▪ *To what extent does your plan to become more physically active/ not become more physically active feel as your own plan?* ▪ *To what extent does your plan to become more physically active/ not become more physically active feel as imposed by others or society?*	1 (not at all as my own plan) – 10 (very much as my own plan) 1 (not imposed at all) – 10 (very much imposed)	0.54^c^	T1
Commitment^b^	▪ *To what extent are you willing to put time, effort and energy in being more physically active?*	1 (not willing at all) – 10 (very willing)		T1, T2
Self-efficacy^b^	▪ *How confident are you that you will succeed in being more physically active?* ▪ *How confident are you that you will succeed in being more physically active when faced with barriers, such as bad weather, lack of time and tiredness?*	1 (not confident at all) – 10 (very confident)	0.85	T1, T2^d^
Satisfaction	▪ *To what extent are you satisfied with you plan to become/not become more physically active?*	1 (not satisfied at all)—10 (very satisfied)		T1
Alignment of choice with personal values	▪ *To what extent does your plan to become/ not become more physically active correspond with what you consider important?*	1 (not at all) – 10 (very much)		T1

*IPAQ* International Physical Activity Questionnaire, *T0* Pre-intervention measurement, *T1* Post-intervention measurement, *T2* Follow-up measurement

^a^Scales were constructed by averaging the responses to the total number of items

^b^Only asked to participants who intended to become more physical active

^c^The second item was reverse coded for the scale; however, due to the low Cronbach’s alpha, the items were analysed separately, without reverse coding

^d^Only the first self-efficacy item was assessed at T2

#### Behavioural outcomes

Physical activity and sitting time were assessed using the short form of the International Physical Activity Questionnaire (IPAQ) [46, 47]. The IPAQ has been shown to have reasonable reliability and validity [48]. It assesses time spent in vigorous and moderate-intensity activities, and walking for at least ten minutes over the last seven days, in addition to time spent sitting. MET-minutes per week were calculated for each physical activity type [42]. A combined total physical activity MET-minutes/week score was computed by summing the vigorous, moderate and walking MET-minutes/week scores. Responses of < 10 min were recoded ‘zero’, and total scores of ≥ 16 h were excluded from the analysis. A dichotomization was also made: the ‘moderate/high active’ category (i.e., ≥ 3 days of vigorous-intensity activity of ≥ 20 min/day, or ≥ 5 days of moderate-intensity activity or walking of ≥ 30 min/day, or ≥ 5 days of any combination of walking, moderate-intensity or vigorous-intensity activities achieving a minimum of 600 MET-minutes/week) versus the ‘low active’ category (i.e., individuals who do not meet the ‘moderate/high active’ criteria) [42]. For sitting time, we excluded cases with ≥ 20 h of daily sitting time from the analysis because such values seemed invalid.

We assessed the perceived increase in physical activity by asking participants at T2 whether they had changed their physical activity behaviour in the past two weeks (yes/no) and, if so, what they had changed. Based on their answers, we categorized participants as ‘more physically active’ or ‘less physically active/no change’.

#### Psychological outcomes

Physical activity intention was assessed dichotomously (yes/no). Intention strength (1 item), the extent to which an autonomous choice was made (2 items), commitment to becoming more physically active (1 item), self-efficacy regarding physical activity (2 items), choice satisfaction (1 item), and the degree to which one’s choice aligns with one’s values (1 item) were assessed using 10-point scales. The degree to which participants made an active choice was assessed by 9 self-constructed items using a 5-point scale, together forming a composite measure (Cronbach’s alpha = 0.77). The first five items of this active choice scale were adapted from the validated Decisional Conflict Scale [49]. All psychological outcomes were assessed post-intervention. During T2, we again assessed intention strength, commitment, and self-efficacy.

### Sociodemographic variables

With participants’ consent, the research agency provided us with sociodemographic information about participants: age, gender, educational level, ethnic background and number of children living at home. At the end of the T1 questionnaire, participants were presented with a list of physical and mental health conditions (e.g., musculoskeletal conditions, cardiovascular conditions, metabolic disorders, cancer, depression) and asked to select conditions that applied to them. Furthermore, an open-ended question was asked (‘Is there anything – related to your physical activity behaviour – that is relevant for the researcher to know?’). Participants who did not complete T1 were asked during T2 to provide a reason for not finishing T1.

### Process evaluation

We performed a process evaluation of the intervention to assess the interventions’ acceptability and identify contextual factors that may have affected the outcomes. In doing so, we followed the Medical Research Council guidance for process evaluations, which distinguishes three key components: Implementation (including dose and reach), Context (i.e., external influences on implementation or effects) and Mechanisms of impact (i.e., mechanisms through which interventions bring about change) [50]. Participants were asked to evaluate the extent to which the intervention was well-organised, understandable, and readable on a 7-point scale. Moreover, they were asked to evaluate the intervention length and the extent to which it was interesting, clear and pleasant to complete on a 5-point scale. Participants were also asked to indicate to which extent the intervention helped them make a (more) deliberate choice about physical activity behaviour. Open-ended questions asked participants whether they had any comments in addition to the evaluation questions or suggestions for improvement.

### Data analysis

Statistical analyses were performed using SPSS for Windows version 26. Differences in physical activity (MET-minutes per week: T2 vs. T0), sitting time (T2 vs. T0), intention strength (T2, and T2 vs. T1), active choice (T1), autonomous choice (T1), commitment (T1, and T2 vs. T1), self-efficacy (T1, and T2 vs. T1), satisfaction (T1), and alignment of choice with personal values (T1) between the GA + , GA, and GI groups and group G (comparison group) were assessed using linear regression analyses; logistic regression analyses were used for physical activity (moderate/high versus low: T2 vs. T0), perceived increase in physical activity (T2) and intention (T1). The four groups were dummy coded and used as independent variables in all analyses. For outcomes assessed at two time points (i.e., at T0/T1 and T2), we used the T2 outcome as the dependent variable and adjusted for the T0/T1 outcome (covariate). Participants who had not completed T0 and T1 were excluded from analyses. Pairwise deletion was used for missing data. In IPAQ data, outliers (i.e., values exceeding 1.5 interquartile ranges below the 25^th^ percentile or above the 75^th^ percentile) were excluded from analyses. A significance level of 0.05 was used. Outcome variables heavily skewed to the right were log-transformed for the regression analyses to meet the assumption of normal distribution.

#### Sensitivity analyses

We conducted multiple sensitivity analyses. In the first set, we examined possible confounding by age and effect modification by gender, educational level (lower/intermediate/higher), and health condition (yes/no) on our primary outcome measures. We used a liberal significance level (alpha = 0.10) for interaction terms [51]. In case of confounding or effect modification, we additionally analysed possible confounding or effect modification on the remaining outcomes. In the second set, we combined group G with group GI and group GA with group GA + and compared their effects on all outcomes. In the third set, we conducted all main analyses without the five GA + participants who had not made action/coping plans because they did not want to become more physically active. Fourthly, we performed a sensitivity analysis for physical activity (MET-minutes per week). In this sensitivity analysis, we excluded the 82 participants who reported – in the open-ended questions – physical or mental health conditions that seemed to severely hinder physical activity, including illness, pain and depression. Three researchers (LL, JJ, OD) independently assessed whether reported health conditions seemed to severely hinder physical activity, and reached a consensus in a meeting. Finally, we examined GA + participants’ planned start dates to become more physically active.

#### Process evaluation

The open-ended questions of the process evaluation were analysed following thematic analysis [52]. First, participants’ answers were inductively coded. Codes concerning the intervention evaluation were subsequently grouped into two pre-defined categories: ‘Function’ (i.e., intervention aim and content) and ‘Form’ (i.e., layout, wording, technical aspects). In the next step, themes were identified by looking for codes that shared a central concept. One researcher (LL) coded all open-ended questions; another researcher (JJ) checked the coding.

## Results

### Study population

**Fig. 2:**
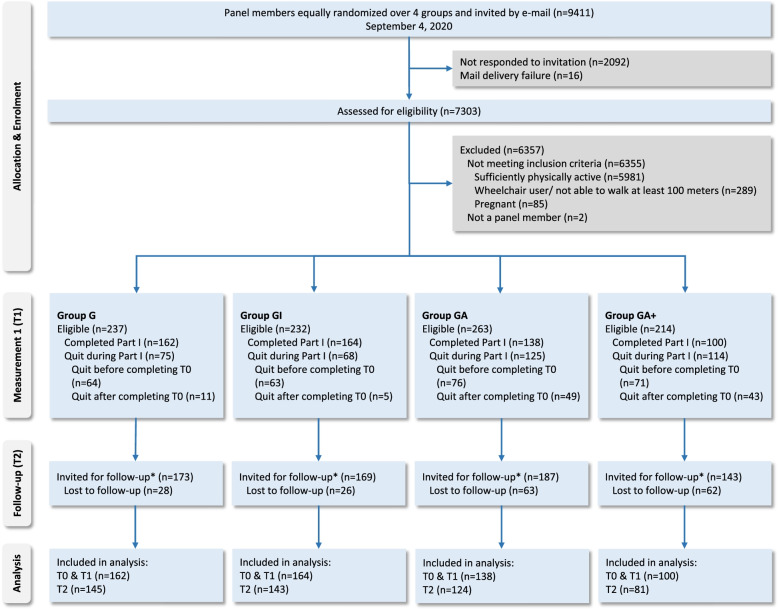
Participant flow diagram

Out of 7,303 panel members, 946 (13%) were eligible for participation. Of these 946 panel members, 564 (60%) fully completed Part I, and 382 (40%) did not complete Part I; 108 of these 382 individuals completed at least T0. The target of 182 participants per group was not met. Out of the 672 participants invited for T2, 493 (73%) responded. Figure 2 presents a corresponding flow diagram.

Group G took the shortest time to complete Part I (median = 8 min) and group GA + the longest (median = 19 min). The mean time interval between Part I and Part II was 18 days (range: 9–33 days).

Table 3 shows participants’ characteristics at baseline. Participants were mostly women (64%) and were on average 49 years (SD = 14.6). Half of our sample (50%) completed higher education, 96% had a Dutch background, 39% lived with children, and 67% reported having a physical or mental health condition. Tables S1 and S2 (Additional file 2) show participants’ baseline characteristics stratified by gender and completion status, respectively.

**Table 3:** Demographics at baseline

**Demographics**	**Group G** (*n* = 162)	**Group GI** (*n* = 164)	**Group GA** (*n* = 138)	**Group GA + ** (*n* = 100)	**Total** (*n* = 564)
Age (years), mean ± SD	50.4 ± 14.3	49.8 ± 14.2	46.6 ± 14.0	50.2 ± 16.3	49.3 ± 14.6
Gender, women, *n* (%)	106 (65.4%)	103 (62.8%)	89 (64.5%)	63 (63.0%)	361 (64.0%)
Educational level, *n* (%)					
Lower	25 (15.4%)	28 (17.1%)	27 (19.6%)	17 (17.0%)	97 (17.2%)
Middle	52 (32.1%)	63 (38.4%)	37 (26.8%)	33 (33.0%)	185 (32.8%)
Higher	85 (52.5%)	73 (44.5%)	74 (53.6%)	50 (50.0%)	282 (50.0%)
Dutch background, *n* (%)	157 (96.9%)	157 (95.7%)	131 (94.9%)	97 (97.0%)	542 (96.1%)
Living with children, *n* (%)	61 (37.7%)	69 (42.1%)	53 (38.4%)	35 (35.0%)	218 (38.7%)
Physical or mental condition, *n* (%)	98 (60.5%)	117 (71.3%)	88 (63.8%)	75 (75.0%)	378 (67.0%)

*SD* standard deviation

### Main analyses

Table 4 shows the results of the regression analyses in which we compared the effects of the GA + , GA, and GI interventions with the guideline only (G) on behavioural and psychological outcomes.

**Table 4:** Regression analyses of the effects of the GA + , GA, and GI interventions compared to the guideline only (group G) on behavioural and psychological outcomes

Outcome	Group	Pre-intervention measurement (T0)		Follow-up measurement (T2) versus T0	
		**Median (IQR)**^a^		**Median (IQR)**^a^	**β [95% CI]**
Physical activity, total MET-minutes per week (*n* = 311)	GA +	640.00 (887.25)		855.00 (1305.50)	β = 1.09 [0.79; 1.51]^b^
	GA	594.00 (713.75)		720.00 (1029.00)	β = 0.98 [0.73; 1.31]^b^
	GI	758.00 (1188.00)		897.00 (1134.00)	β = 1.16 [0.88; 1.54]^b^
	G	672.00 (993.00)		844.00 (1158.00)	
		**Mean (SD) or N (%)**	**β or OR [95% CI]**	**Mean (SD) or N (%)**	**β or OR [95% CI]**
Physical activity, category ‘moderate/high’^c^ (*n* = 477)	GA +	46 (51.7%)		34 (35.4%)	OR = 0.62 [0.35; 1.11]
	GA	46 (36.5%)		53 (40.8%)	OR = 0.93 [0.55; 1.57]
	GI	58 (40.8%)		56 (36.8%)	OR = 0.87 [0.53; 1.45]
	G	63 (43.4%)		65 (43.6%)	
Sitting time, minutes per day (*n* = 408)	GA +	522.34 (217.77)		519.35 (218.66)	β = 17.84 [-25.66; 61.33]
	GA	525.49 (199.00)		504.25 (224.45)	β = 2.42 [-37.22; 42.05]
	GI	507.02 (190.33)		477.57 (193.45)	β = -11.07 [-48.93; 26.78]
	G	497.45 (199.77)		482.22 (200.55)	
Perceived increase in physical activity (*n* = 499)	GA +			37 (44.0%)	OR = 2.61** [1.44; 4.72]
	GA			36 (28.3%)	OR = 1.19 [0.67; 2.10]
	GI			38 (26.6%)	OR = 1.06 [0.60; 1.87]
	G			36 (24.8%)	
		**Post-intervention measurement (T1)**		**Follow-up measurement (T2) versus T1**	
Intention (*n* = 564)	GA +	87 (87.0%)	OR = 1.65 [0.82; 3.32]		
	GA	108 (78.3%)	OR = 0.89 [0.51; 1.55]		
	GI	135 (82.3%)	OR = 1.15 [0.66; 2.00]		
	G	130 (80.2%)			
Intention strength (T1: *n* = 564; T2: *n* = 491)	GA +	6.32 (2.40)	β = 0.41 [-0.14; 0.97]	6.23 (2.31)	β = 0.08 [-0.39; 0.56]
	GA	6.17 (2.23)	β = 0.27 [-0.24; 0.77]	6.18 (2.30)	β = 0.12 [-0.31; 0.55]
	GI	6.54 (2.07)	β = 0.64* [0.15; 1.12]	6.45 (2.21)	β = 0.16 [-0.25; 0.58]
	G	5.91 (2.26)		5.90 (2.08)	
Active choice (*n* = 564)	GA +	3.66 (0.50)	β = 0.09 [-0.03; 0.22]		
	GA	3.73 (0.49)	β = 0.16** [0.04; 0.27]		
	GI	3.70 (0.51)	β = 0.13* [0.02; 0.24]		
	G	3.57 (0.49)			
Autonomy: ‘Own choice’, (*n* = 564)	GA +	6.83 (2.16)	β = 0.22 [-0.30; 0.74]		
	GA	6.80 (1.91)	β = 0.19 [-0.28; 0.66]		
	GI	7.11 (2.04)	β = 0.50* [0.05; 0.95]		
	G	6.61 (2.16)			
Autonomy: ‘Imposed choice’ (*n* = 564)	GA +	4.48 (2.57)	β = 0.26 [-0.34; 0.87]		
	GA	4.61 (2.51)	β = 0.39 [-0.16; 0.95]		
	GI	4.34 (2.41)	β = 0.12 [-0.41; 0.65]		
	G	4.22 (2.31)			
Commitment (T1: *n* = 460; T2: *n* = 401)	GA +	7.26 (1.54)	β = 0.44* [0.04; 0.84]	6.33 (2.11)	β = 0.19 [-0.39; 0.77]
	GA	7.09 (1.25)	β = 0.26 [-0.11; 0.63]	6.17 (2.20)	β = 0.14 [-0.39; 0.67]
	GI	7.22 (1.48)	β = 0.39* [0.04; 0.74]	6.43 (2.22)	β = 0.32 [-0.18; 0.83]
	G	6.82 (1.52)		5.87 (2.03)	
Self-efficacy (composite score) (*n* = 460)	GA +	5.74 (1.62)	β = 0.26 [-0.22; 0.74]		
	GA	5.81 (1.75)	β = 0.32 [-0.11; 0.76]		
	GI	5.85 (1.78)	β = 0.37 [-0.05; 0.79]		
	G	5.48 (1.72)			
Self-efficacy (single item) (*n* = 401)	GA +	6.07 (1.75)		5.51 (2.23)	β = 0.16 [-0.37; 0.70]
	GA	6.15 (1.82)		5.56 (2.07)	β = 0.17 [-0.31; 0.66]
	GI	6.21 (1.87)		5.81 (2.01)	β = 0.38 [-0.09; 0.84]
	G	5.94 (1.85)		5.28 (1.95)	
Satisfaction (*n* = 564)	GA +	6.57 (1.90)	β = 0.32 [-0.16; 0.80]		
	GA	6.37 (1.99)	β = 0.12 [-0.32; 0.55]		
	GI	6.53 (1.88)	β = 0.28 [-0.14; 0.70]		
	G	6.25 (1.92)			
Alignment of choice with personal values (*n* = 564)	GA +	7.16 (1.79)	β = 0.28 [-0.17; 0.74]		
	GA	7.08 (1.82)	β = 0.20 [-0.21; 0.62]		
	GI	7.27 (1.93)	β = 0.39 [-0.00; 0.79]		
	G	6.88 (1.69)			

*IQR* interquartile range, *CI* confidence interval, *MET* metabolic equivalent of task, *SD* standard deviation, β regression coefficient, *OR* odds ratio

^*^P < .05

^**^P < .01

^a^The median and (IQR) are reported as the distribution is skewed to the right

^b^The results were log transformed for the analysis (using the natural logarithm) and subsequently back transformed

^c^The ‘moderate/high’ category was compared to the ‘low’ category

#### Behavioural outcomes

Physical activity (both as a continuous variable and dichotomized variable) and sitting time did not significantly differ between groups over time. However, a significantly higher proportion of participants in group GA + (44%) reported a perceived increase in physical activity between T0 and T2 than participants in group G (25%); β = 2.61, 95%CI:1.44;7.72.

#### Psychological outcomes

Intention to become more physically active at T1 did not significantly differ between groups. Regarding intention strength, we found a significantly stronger intention in group GI compared to group G at T1; β = 0.64, 95%CI:0.15;1.12. Furthermore, the extent to which participants made an active choice regarding physical activity was significantly higher in groups GA and GI compared to group G. Concerning autonomy, we found that group GI perceived their choice significantly more as their ‘own choice’ compared to group G; however, no significant differences were found for ‘imposed choice’. Commitment to becoming more physically active was significantly higher in groups GA + and GI compared to group G at T1. Self-efficacy did not significantly differ between groups at T1 or T2. Scores on satisfaction and ‘alignment of choice with personal values’ were not significantly different between groups.

### Sensitivity analyses

We found no confounding by age and no effect modification by educational level in the first set of sensitivity analyses. However, we found effect modification by gender on physical activity (dichotomous outcome), sitting time, intention, intention strength, and commitment, and effect modification of health condition (i.e., at least one health condition vs. no health condition) on physical activity (continuous outcome), sitting time, commitment, self-efficacy, and the perceived increase in physical activity. Table S3 (Additional file 2) shows the subgroup analyses for these outcomes. Looking at the differences between group GA + and G, it appeared that significantly more women in group GA + intended to become more physically active (OR = 54.40, 95% CI:1.21;16.36) and that their intention strength was significantly higher at T1 (β = 0.88, 95%CI:0.23;1.53). Concerning health conditions, we observed no clear pattern; participants with or without a health condition did not seem to benefit more from the GA + intervention than from the guideline only (G).

The second set of analyses did not show any significant effects. The results of the third and fourth set of analyses were similar to the results of the main analyses. Finally, we found that among the GA + participants who planned a start date to become more physically active (*n* = 92), 67% planned to start within 14 days, while 33% planned to start later.

### Process evaluation

#### Implementation

As shown in Fig. 2, participants in groups GA + and GA more frequently quit participation during Part I, and a higher percentage in these groups was lost to follow-up compared to groups G and GI. Participants who quit during Part I frequently mentioned personal circumstances (*n* = 17) – mostly ‘lack of time’ – as the reason. However, 23 participants mentioned they had quit Part I because of the intervention or questionnaires; they thought it was not interesting, difficult to understand, insufficiently applicable to their situation, too long, too confronting, or too demotivating. Twelve participants stated that they had forgotten why they had quit.

#### Context

Table S4 (Additional file 3) shows participants’ general quantitative evaluation of the intervention. Participants' evaluation of the intervention length and the degree to which it was interesting, clear, pleasant to complete, well-organized, understandable and readable was comparable across groups. However, participants in group GA + had the lowest ratings on ‘clearness’ (M = 3.87, SD = 1.10) and ‘pleasantness’ (M = 3.70, SD = 1.11), and somewhat more often indicated that it was too long (M = 3.30, SD = 0.77) compared to the other groups. Finally, participants’ evaluation of the intervention form (Table S5, Additional file 3) showed that some participants thought that items were confusing, complicated, too long, or too similar.

#### Mechanisms of impact

Table S6 (Additional file 3) shows to what extent participants thought the intervention had supported them in making a (more) deliberate choice about their physical activity behaviour. Participants in group GA + rated the degree to which the intervention had increased awareness about their physical activity behaviour with 4.64 (SD = 1.50) and the degree to which it had helped make a (more) deliberate choice with 4.28 (SD = 1.46), both on a 7-point scale. Mean ratings in the other groups were comparable. Groups GA + and GA rated the active choice intervention exercises on average 6.06 (range: 5.93–6.24) on a 10-point scale.

Our analysis of the open-ended questions concerning the evaluation of the intervention (Table S5, Additional file 3) showed that some participants were explicitly positive; they indicated that it made them more aware of their behaviour and health or that it was clarifying, pleasant to complete or motivating. However, some were explicitly negative; according to them, the intervention did not support or motivate them, or the topic ‘physical activity’ was negatively evaluated. Two participants thought that the tone was too paternalistic. Furthermore, participants often mentioned that they were already aware of their behaviour or the provided information. Positive and negative evaluations were equally common across groups.

#### Suggestions for improvement

Participants had multiple suggestions for improvement, including taking into account their situation – especially physical impairments; communicating more benefits of physical activity; using more visual examples and less text; providing advice about increasing physical activity; focussing more on the fun of physical activity; and simplifying wording of items (Table S5, Additional file 3).

## Discussion

The results of this pre-test post-test web-based experiment showed that promoting an active choice process (i.e., the GA + intervention) did not significantly increase intention and self-reported physical activity behaviour compared to providing a physical activity guideline only (i.e., the more passive choice group ‘G’). However, the GA + intervention did significantly increase commitment immediately after the intervention as well as the perception of an increase in physical activity at 2–4 weeks follow-up compared to group G. An unexpected finding was that only GI participants – who received the physical activity guideline and additional information – reported a significantly more active and autonomous choice and more commitment than those provided with the guideline only (group G). Finally, the sensitivity analyses showed that gender and health condition were effect modifiers on several outcomes.

Although GA + participants’ physical activity levels did not significantly increase compared to group G according to the IPAQ data, a significantly greater proportion of GA + participants reported a perceived increase in physical activity at follow-up. Their perceived increase in physical activity may have been too small to be detected by the IPAQ. GA + participants also reported significantly more commitment to physical activity immediately after the intervention compared to participants in group G. Based on the literature, this positive effect on commitment seems to be attributable to the combination of the value-clarification exercise and action- and coping exercises [9, 11, 13, 14]. More specifically, it is thought that the value-clarification exercise elicited cognitive dissonance in GA + participants – due to increased awareness of a discrepancy between current behaviour and ‘the value of health’ – and that participants resolved this by making concrete action- and coping plans.

The GA + intervention – which was theory-based and contained components previously shown to be effective – did not significantly improve overall intention nor physical activity behaviour. First of all, this may be explained by a lack of professional guidance and follow-up support in the intervention. Previous interventions containing active choice components (i.e., based on MI, the DVM, ACT, or containing implementation intentions) that were shown to increase physical activity [17–20] and/or physical fitness [21, 22] were usually provided face-to-face by a professional and also included follow-up support. Moreover, action and coping planning have been suggested to result in better outcomes if professional assistance is provided, especially in middle-aged and older adults [53]. Our study was web-based; although internet-delivered interventions generally have the advantage of reaching a large number of individuals at an acceptable cost [54], their effects on physical activity are generally relatively small [55]. Given the fact that web-based interventions with a longer duration and a higher number of intervention contacts produce larger effect sizes [55], it seems that professional guidance and follow-up support are also key aspects for web-based interventions.

Secondly, the lack of effects of the GA + intervention on most outcomes could be related to its form. More specifically, the results of our process evaluation as well as the relatively high drop-out rates in the active choice groups seem to point out that completing the intervention required much time and cognitive effort. Previous research has demonstrated that decision makers understand information better and make better choices if less cognitive effort is required [56]. Cognitive demands may be reduced by considering participants’ recommendations of using simple, understandable language, less text and more visual examples.

Thirdly, the lack of effects on many outcomes may be explained by the fact that the GA + intervention insufficiently considered individual and intergroup differences. Since the GA + intervention was not tailored to prior knowledge and physical abilities, participants often mentioned that they were already aware of the provided information and that the intervention insufficiently considered physical impairments. Previous research suggests that health-promoting messages and interventions are more effective if tailored to individual needs or targeted to specific subgroups because such messages command greater attention, contain less redundant information, and enhance the personal relevance of information [57–59]. Regarding differences between subgroups, we found that the intervention differently impacted men versus women. Web-based interventions may especially interest women, as women are more likely than men to participate in web-based physical activity interventions [55]. Men may benefit more from non-web-based gender-sensitised approaches (e.g., [60, 61]). Our intervention also differentially impacted individuals with a health condition versus those without a health condition. Individuals with health conditions may benefit from autonomy and self-efficacy support to aid disease self-management, including lifestyle change [62–64]. In sum, future active choice interventions are advised to adopt a more gender-sensitive approach that also considers physical and mental health conditions.

The positive effects of the GI intervention on participants’ autonomy and the degree to which they made an active choice may be due to the fact that GI participants were informed about possible advantages and disadvantages and barriers to physical activity, whereas participants in control group G did not receive this information. Consequently, participants in group GI may have made a somewhat more informed choice compared to participants in group G.

### Strengths and limitations

Important strengths include the randomized controlled design of the study, the theoretical basis of the intervention components, and the fact that we included a process evaluation. These strengths contributed to a better understanding of findings [65, 66]. However, our results should be interpreted with caution because the intended sample size was not reached, and multiple statistical tests were performed, increasing the likelihood of a Type 1 error [67]. This is of particular concern for the stratified analyses, as group sizes were even smaller. A second limitation is that self-report measures were used to assess behavioural outcomes. Prior studies comparing self-reported IPAQ data with accelerometer-measured data showed that participants tend to over-report vigorous physical activity and underreport sitting time [40, 68]. Although more reliable results would have been obtained by using accelerometers, we have deliberately used the IPAQ questionnaire for feasibility reasons. Thirdly, our study lacked long-term follow-up measurements. Therefore, our results may not reflect the physical activity changes of participants who planned to start becoming more physically active *after* two weeks – which was the case for 33% of GA + participants.

## Conclusions

Overall, our results do not show a beneficial effect of promoting an active choice process regarding physical activity on physically inactive adults’ physical activity intentions and behaviours compared to providing a physical activity guideline only. However, compared to the guideline only, the intervention condition that promoted the most active choice process significantly increased inactive adults’ commitment to physical activity and their perception of an increase in physical activity at follow-up. Interestingly, our results suggest that the active choice intervention differently impacts men versus women and individuals with a health condition versus those without a health condition. Further research is needed to determine whether and how active choice interventions that are gender-sensitized, consider health conditions and include professional guidance and follow-up support can effectively increase physical activity in inactive adults.

## Supplementary Information

**Additional file 1. **GA+ intervention.

**Additional file 2: Table S1. **Demographics of men and women at baseline. **Table S2. **Demographics of completers and non-completers of Part I. **Table S3. **Regression analyses of the effects of GA+, GA, andGI interventions compared to the guideline only (group G) on behavioural and psychological outcomes stratified by gender and/or by physical or mentalhealth condition.

**Additional file 3: Table S4. **Participants' general quantitative evaluation of the interventionand questionnaire. **Table S5. **Qualitative analysis of participants' evaluation ofthe function and form of the intervention and questionnaire and participants’suggestions for improvement. **Table S6. **Participants’ quantitative evaluation of the extentto which the intervention supported deliberate decision making about physicalactivity.

## Data Availability

The datasets used and analysed during the current study are available from the corresponding author on reasonable request.

## Abbreviations

MI: Motivational interviewing
DVM: Disconnected Values Model
ACT: Acceptance and Commitment Therapy
BCT: Behaviour Change Technique
IPAQ: International Physical Activity Questionnaire
PA: Physical activity
MET: Metabolic equivalent of task
IQR: Interquartile range
CI: Confidence interval
SD: Standard deviation
β: Regression coefficient
OR: Odds ratio
