# Supplementary material for: Promoting an active choice among physically inactive adults: a randomised web-based four-arm experiment
Source: Int J Behav Nutr Phys Act. 2022 Apr 27;19:49. doi: 10.1186/s12966-022-01288-y (PMC9043878; doi:10.1186/s12966-022-01288-y)
Supplement: Supplementary file 1 — Additional file 1. GA+ intervention. [file 12966_2022_1288_MOESM1_ESM.docx]

**Additional File 1: GA+ intervention**

**Advantages and disadvantages**

You answered questions about your current physical activity behaviour, such as walking, cycling, stair walking and exercise.

In the current exercise, we ask you to follow the steps below and to complete the four boxes. Examples can be found in the information buttons. Please use a separate line for each advantage/ disadvantage.

**Step 1:** In the light blue box (top left), describe the advantages you experience from your current physical activity behaviour.

**Step 2:** In the purple box (top right), describe the disadvantages you experience from your current physical activity behaviour.

**Step 3:** In the orange box (bottom left), describe the disadvantages you could experience if you would engage more in physical activity.

**Step 4:** In the dark blue box (bottom right), describe the advantages you could experience if you would engage more in physical activity.

**Disadvantages of my current physical activity behaviour:**

□ ____________________________

□ ____________________________

□ ____________________________

□ ____________________________

□ ____________________________

□ ____________________________

**Advantages of my current physical activity behaviour:**

□ ____________________________

□ ____________________________

□ ____________________________

□ ____________________________

□ ____________________________

□ ____________________________

**Examples [info button]**

*- It does not cost me extra money*

*- I have time for relaxation*

**Examples [info button]**

*- I have an unhealthy weight*

*- Increased risk of cardiovascular disease*

**Advantages of ‘more physical activity’**

□ ____________________________

□ ____________________________

□ ____________________________

□ ____________________________

□ ____________________________

□ ____________________________

**Disadvantages of ‘more physical activity’**

□ ____________________________

□ ____________________________

□ ____________________________

□ ____________________________

□ ____________________________

□ ____________________________

**Examples [info button]**

*- I improve my fitness*

*- It gives me energy*

**Examples [info button]**

*- It takes time that I want to spend on other things*

*- It makes me sweat*

**Please take a closer look at the advantages and disadvantages you have entered in the 4 boxes. Select the 3 that you consider most important (you must choose a total of 3 advantages/ disadvantages from the 4 boxes).**

**< Next page >**

**What I consider important**

In making choices, it can be helpful to consider what is important to you.

How **important** are the following elements to you in your life? Please indicate the importance of each element on a scale from 1 (not important) to 10 (very important).

|  | 1 (not important) | 2 | 3 | 4 | 5 | 6 | 7 | 8 | 9 | 10 (very important) |
| --- | --- | --- | --- | --- | --- | --- | --- | --- | --- | --- |
|  |  |  |  |  |  |  |  |  |  |  |
| **Health** | □ | □ | □ | □ | □ | □ | □ | □ | □ | □ |
|  |  |  |  |  |  |  |  |  |  |  |
| **Responsibility** | □ | □ | □ | □ | □ | □ | □ | □ | □ | □ |
|  |  |  |  |  |  |  |  |  |  |  |
| **Performance** (e.g. at work) | □ | □ | □ | □ | □ | □ | □ | □ | □ | □ |
|  |  |  |  |  |  |  |  |  |  |  |
| **Pleasure** | □ | □ | □ | □ | □ | □ | □ | □ | □ | □ |
|  |  |  |  |  |  |  |  |  |  |  |
| **Family** | □ | □ | □ | □ | □ | □ | □ | □ | □ | □ |
|  |  |  |  |  |  |  |  |  |  |  |
| **Friendships** | □ | □ | □ | □ | □ | □ | □ | □ | □ | □ |
|  |  |  |  |  |  |  |  |  |  |  |
| **Balance** (e.g. between work and private life) | □ | □ | □ | □ | □ | □ | □ | □ | □ | □ |

**< Next page >**

How much time, effort and energy did you actually spend on each of these elements in the past year? Please indicate this for each element on a scale from 1 (very little) to 10 (very much).

|  | 1 (very little) | 2 | 3 | 4 | 5 | 6 | 7 | 8 | 9 | 10 (very much) |
| --- | --- | --- | --- | --- | --- | --- | --- | --- | --- | --- |
|  |  |  |  |  |  |  |  |  |  |  |
| **Health** | □ | □ | □ | □ | □ | □ | □ | □ | □ | □ |
|  |  |  |  |  |  |  |  |  |  |  |
| **Responsibility** | □ | □ | □ | □ | □ | □ | □ | □ | □ | □ |
|  |  |  |  |  |  |  |  |  |  |  |
| **Performance** (e.g. at work) | □ | □ | □ | □ | □ | □ | □ | □ | □ | □ |
|  |  |  |  |  |  |  |  |  |  |  |
| **Pleasure** | □ | □ | □ | □ | □ | □ | □ | □ | □ | □ |
|  |  |  |  |  |  |  |  |  |  |  |
| **Family** | □ | □ | □ | □ | □ | □ | □ | □ | □ | □ |
|  |  |  |  |  |  |  |  |  |  |  |
| **Friendships** | □ | □ | □ | □ | □ | □ | □ | □ | □ | □ |
|  |  |  |  |  |  |  |  |  |  |  |
| **Balance** (e.g. between work and private life) | □ | □ | □ | □ | □ | □ | □ | □ | □ | □ |

**< Next page >**

Below is overview of your answers. Please compare your score on *importance* with your score on the amount of *time, effort and energy* you have spent on it for **Health.** Check whether there is a difference between those scores.

The meaning of the scores is:

- Importance (blue): 1 = not important, 10 = very important
- Time, effort, energy (orange): 1= very little, 10 = very much

|  |  | 1 | 2 | 3 | 4 | 5 | 6 | 7 | 8 | 9 | 10 |
| --- | --- | --- | --- | --- | --- | --- | --- | --- | --- | --- | --- |
|  |  |  |  |  |  |  |  |  |  |  |  |
| **Health** | Importance | □ | □ | □ | □ | □ | □ | □ | □ | □ | □ |
|  | Time, effort, energy | □ | □ | □ | □ | □ | □ | □ | □ | □ | □ |
|  |  |  |  |  |  |  |  |  |  |  |  |

Would you like to spend more time, effort and energy on your health?

□ Yes

□ No

**< Next page >**

In addition to the importance of your health, other elements can influence your physical activity behaviour as well; either directly or indirectly. An overview of your answers for the other elements is provided below.

The meaning of the scores is:

- Importance (blue): 1 = not important, 10 = very important
- Time, effort, energy (orange): 1= very little, 10 = very much

| **Responsibility** | Importance | □ | □ | □ | □ | □ | □ | □ | □ | □ | □ |
| --- | --- | --- | --- | --- | --- | --- | --- | --- | --- | --- | --- |
|  | Time, effort, energy | □ | □ | □ | □ | □ | □ | □ | □ | □ | □ |
|  |  |  |  |  |  |  |  |  |  |  |  |
| **Performance** (e.g. at work) | Importance | □ | □ | □ | □ | □ | □ | □ | □ | □ | □ |
|  | Time, effort, energy | □ | □ | □ | □ | □ | □ | □ | □ | □ | □ |
|  |  |  |  |  |  |  |  |  |  |  |  |
| **Pleasure** | Importance | □ | □ | □ | □ | □ | □ | □ | □ | □ | □ |
|  | Time, effort, energy | □ | □ | □ | □ | □ | □ | □ | □ | □ | □ |
|  |  |  |  |  |  |  |  |  |  |  |  |
| **Family** | Importance | □ | □ | □ | □ | □ | □ | □ | □ | □ | □ |
|  | Time, effort, energy | □ | □ | □ | □ | □ | □ | □ | □ | □ | □ |
|  |  |  |  |  |  |  |  |  |  |  |  |
| **Friendships** | Importance | □ | □ | □ | □ | □ | □ | □ | □ | □ | □ |
|  | Time, effort, energy | □ | □ | □ | □ | □ | □ | □ | □ | □ | □ |
|  |  |  |  |  |  |  |  |  |  |  |  |
| **Balance** (e.g. between work and private life) | Importance | □ | □ | □ | □ | □ | □ | □ | □ | □ | □ |
|  | Time, effort, energy | □ | □ | □ | □ | □ | □ | □ | □ | □ | □ |

Please indicate for each element to what extent it affects your physical activity behaviour on a scale from 1 (not at all) to 10 (very much).

|  | 1 (not at all) | 2 | 3 | 4 | 5 | 6 | 7 | 8 | 9 | 10 (very much) |
| --- | --- | --- | --- | --- | --- | --- | --- | --- | --- | --- |
|  |  |  |  |  |  |  |  |  |  |  |
| **Responsibility** | □ | □ | □ | □ | □ | □ | □ | □ | □ | □ |
|  |  |  |  |  |  |  |  |  |  |  |
| **Performance** (e.g. at work) | □ | □ | □ | □ | □ | □ | □ | □ | □ | □ |
|  |  |  |  |  |  |  |  |  |  |  |
| **Pleasure** | □ | □ | □ | □ | □ | □ | □ | □ | □ | □ |
|  |  |  |  |  |  |  |  |  |  |  |
| **Family** | □ | □ | □ | □ | □ | □ | □ | □ | □ | □ |
|  |  |  |  |  |  |  |  |  |  |  |
| **Friendships** | □ | □ | □ | □ | □ | □ | □ | □ | □ | □ |
|  |  |  |  |  |  |  |  |  |  |  |
| **Balans** (bijv. tussen werk en privé) | □ | □ | □ | □ | □ | □ | □ | □ | □ | □ |

**< Next page >**

**Change**

Sufficient physical activity contributes to good health.

The Dutch health council (Gezondheidsraad) has developed the following physical activity recommendation for adults:

**DUTCH PHYSICAL ACTIVITY RECOMMENDATION**

- - **Physical activity is good, more physical activity is better.**
  - **Engage in at least 150 minutes of moderate level physical activity per week, such as walking and cycling, spread over multiple days. Longer, more frequent and/or more intensive physical activity provides additional health benefits.**
  - **Engage in muscle-^1^ and bone-^2^ strengthening activities at least twice a week, for elderly combined with balance exercises^3^.**
  - **And: Avoid prolonged sitting.**

^1^ ***Muscle-strengthening activities*** *are aimed at improving the strength, endurance, and size of skeletal muscles. Examples are strength training exercises and endurance activities such as walking, cycling, dancing and swimming.*

^2^ ***Bone-strengthening activities*** *consist of* *strength training and activities that charge the body with its own weight, such as jumping, stair walking, walking, running and dancing.*

^3^ ***Balance exercises*** *are aimed at improving balance while standing or moving, for instance: standing on one leg or picking up an object from the ground.*

**< Next page >**

By making small changes in your daily behaviour, you can start to become more physically active. Others have used the following strategies to become more physically active:

**STRATEGIES**

- Going for a walk during lunch break
- Always going by bike for distances up to 5km
- Taking stairs more often, instead of the elevator/escalator
- Walking the neighbour’s dog
- Walking or exercising together with a friend or family member
- Start doing volunteer work, such as walking with elderly in wheelchairs
- Buying a pedometer and monitoring the number of steps
- Moving with music
- Doing squats while toothbrushing

**Would you like to engage more in physical activity?**

□ Yes

□ Maybe

□ No *[Participant is directly linked to ‘My barriers’ and then directly to the questionnaire; he/she does not complete the exercises ‘How to change’ and ‘Coping with barriers’]*

**< Next page >**

**How to change**

It is helpful to think in advance about how, where and when you would like to be physically active.

**What kind of physical activity would you like to engage in?** Make sure that your wish is *feasible.*

*_______________________________________________________________________________*

**How often would you like to engage in this?** *(number of times per week)*

*_______________________________________________________________________________*

**Where would you like to do this?** *(location/situation)*

*_______________________________________________________________________________*

**When exactly would you like to do this?** *(which days and which moment of the day?)*

*_______________________________________________________________________________*

**When would you like to start this?** *(which date?)*

*_______________________________________________________________________________*

**< Next page >**

**My barriers**

You may encounter barriers both when maintaining your current physical activity behaviour as well as when choosing to become more physically active. Barriers can be external factors (such as bad weather), but also internal factors (such as tiredness).

**What barriers keep you from being physically active or becoming more physically active?**

Please first think about this for yourself. Next, you can click the button to view examples of barriers that are often mentioned by others.

**Examples of barriers [info button]**

- Tiredness, lack of energy
- Lack of motivation
- Lack of inspiration (e.g. with regard to the type of physical activity)
- Physical complaints/pain
- Bad weather conditions
- High work pressure
- Lack of practical possibilities
- No childcare for the children
- Little support from others
- Others in my environment are not physically active either

**My barriers:**

**1.** _____________________________________

**2.** _____________________________________

**3.** _____________________________________

**4.** _____________________________________

**5.** _____________________________________

**6.** _____________________________________

**< Next page >**

**Coping with barriers**

If you intend to become more physically active, it is helpful to think in advance about how to cope with barriers.

**You wrote down barriers in the previous exercise. For each of these barriers, think of what you could do to cope with it. Please write down your plans below.**

The more precise, concrete and personal your plans are, the more they can help you.

Below are examples of plans that others have made to cope with barriers.

**My barrier:** [Show first answer from previous exercise]

**EXAMPLES OF PLANS TO COPE WITH BARRIERS**

- If it’s raining, then I’ll cycle to work in my rain suit (and leave the car at home).
- If I can’t exercise in the evening because I’m busy at work, then I’ll exercise on Saturday mornings.
- If I can’t engage in physical activity outside because I have to watch the kids, then I’ll do something active at home together with the kids.

**My plan to cope with this barrier:**

_____________________________________________

_____________________________________________

**My barrier:** [Show second answer from previous exercise]

**My plan to cope with this barrier:**

_____________________________________________

_____________________________________________

**My barrier:** [Show third answer from previous exercise]

**My plan to cope with this barrier:**

_____________________________________________

_____________________________________________

**My barrier:** [Show fourth answer from previous exercise]

**My plan to cope with this barrier:**

_____________________________________________

_____________________________________________

**My barrier:** [Show fifth answer from previous exercise]

**My plan to cope with this barrier:**

_____________________________________________

_____________________________________________

**My barrier:** [Show sixth answer from previous exercise]

**My plan to cope with this barrier:**

_____________________________________________
