# Supplementary material for: Promoting an active choice among physically inactive adults: a randomised web-based four-arm experiment
Source: Int J Behav Nutr Phys Act. 2022 Apr 27;19:49. doi: 10.1186/s12966-022-01288-y (PMC9043878; doi:10.1186/s12966-022-01288-y)
Supplement: Supplementary file 2 — Additional file 2: Table S1. Demographics of men and women at baseline. Table S2. Demographics of completers and non-completers of Part I. Table S3. Regression analyses of the effects of GA+, GA, andGI interventions compared to the guideline only (group G) on behavioural and psychological outcomes stratified by gender and/or by physical or mentalhealth condition. [file 12966_2022_1288_MOESM2_ESM.docx]

**Additional file 2: Supplementary tables – Results of stratified regression analyses and demographics**

**Landais et al. *Promoting an Active Choice among Physically Inactive Adults: A Randomised Web-Based Four-Arm Experiment***

**Table S1.** Demographics of men and women at baseline

| **Demographics** | **Group G** | | **Group GI** | | **Group GA** | | **Group GA+** | |
| --- | --- | --- | --- | --- | --- | --- | --- | --- |
|  | **Men** (*n*=56) | **Women** (*n*=106) | **Men** (*n*=61) | **Women** (*n*=103) | **Men** (*n*=49) | **Women** (*n*=89) | **Men** (*n*=37) | **Women** (*n*=63) |
| Age (years), mean ± SD | 54.8  ± 12.9 | 48.0  ± 14.5 | 53.0  ± 14.2 | 47.9  ± 13.9 | 48.1  ± 12.6 | 45.8  ± 14.8 | 54.6  ± 13.4 | 47.6  ± 17.4 |
| Educational level, *n* (%) |  |  |  |  |  |  |  |  |
| Lower | 10 (18%) | 15 (14%) | 7 (12%) | 21 (20%) | 9 (18%) | 18 (20%) | 4 (11%) | 13 (21%) |
| Middle | 15 (27%) | 37 (35%) | 25 (41%) | 38 (37%) | 14 (29%) | 23 (26%) | 9 (24%) | 24 (38%) |
| Higher | 31 (55%) | 54 (51%) | 29 (48%) | 44 (43%) | 26 (53%) | 48 (54%) | 24 (65%) | 26 (41%) |
| Dutch background, *n* (%) | 55 (98%) | 102 (96%) | 59 (97%) | 98 (95%) | 48 (98%) | 83 (93%) | 37 (100%) | 60 (95%) |
| Living with children, *n* (%) | 17 (30%) | 44 (42%) | 26 (43%) | 42 (42%) | 21 (43%) | 32 (36%) | 16 (43%) | 19 (30%) |
| Physical or mental condition, *n* (%) | 33 (59%) | 65 (61%) | 40 (66%) | 77 (75%) | 26 (53%) | 62 (70%) | 24 (65%) | 51 (81%) |

Abbreviations: *SD* standard deviation

**Table S2.** Demographics of completers and non-completers of Part I

| **Demographics** | **Completers** (*n*=564) | **Non-completers** (*n*=108) |
| --- | --- | --- |
| Age (years), mean ± SD | 49.3 ± 14.6 | 54.3 ± 14.9 |
| Gender, women, *n* (%) | 361 (64.0%) | 72 (66.7%) |
| Educational level, *n* (%) |  |  |
| Lower | 97 (17.2%) | 26 (24.1%) |
| Middle | 185 (32.8%) | 43 (39.8%) |
| Higher | 282 (50.0%) | 39 (36.1%) |
| Dutch background *n* (%) | 542 (96.1%) | 105 (97.2%) |
| Living with children *n* (%) | 218 (38.7%) | 41 (38.0%) |

Abbreviations: *SD* standard deviation

**Table S3.** Regression analyses of the effects of GA+, GA, and GI interventions compared to the guideline only (group G) on behavioural and psychological outcomes stratified by gender and/or by physical or mental health condition.

| **Outcome** | **Physical/mental health condition** | **Gender** | **Group** | **Pre-intervention measurement (T0)** | | **Follow-up measurement (T2) versus T0** | |
| --- | --- | --- | --- | --- | --- | --- | --- |
|  |  |  |  | **Median (IQR)**^a^ |  | **Median (IQR)**^a^ | **β [95% CI]** |
|  |  |  |  |  |  |  |  |
| Physical activity,  total MET-minutes per week^b^ | Health condition (n=200) |  | GA+ | 483.75 (922.75) |  | 801.00 (1568.63) | β = 1.43 [0.94; 2.18]^b^ |
|  |  |  | GA | 558.00 (571.25) |  | 834.00 (1264.50) | β = 1.35 [0.90; 2.01]^b^ |
|  |  |  | GI | 873.00 (1242.00) |  | 874.00 (1059.00) | β = 1.45 [0.99; 2.10]^b^ |
|  |  |  | G | 560.00 (915.00) |  | 597.00 (871.50) |  |
|  |  |  |  |  |  |  |  |
|  | No health condition (n=111) |  | GA+ | 990.00 (967.50) |  | 960.00 (1092.00) | β = 0.71 [0.43; 1.20]^b^ |
|  |  |  | GA | 919.00 (953.38) |  | 594.00 (950.63) | β = **0.59* [-0.39; 0.90]**^b^ |
|  |  |  | GI | 678.00 (1109.50) |  | 918.00 (1200.00) | β = 0.84 [0.55; 1.28]^b^ |
|  |  |  | G | 789.00 (928.50) |  | 1386.00 (1174.50) |  |
|  |  |  |  |  |  |  |  |
|  |  |  |  | **Mean (SD) or N (%)** | **β or OR [95% CI]** | **Mean (SD) or N (%)** | **β or OR [95% CI]** |
| Physical activity,  category ‘moderate/high’^c^ |  | Men  (n=171) | GA+ | 15 (42.9%) |  | 12 (32.4%) | OR = 0.14 [0.17; 1.16] |
|  |  |  | GA | 13 (29.5%) |  | 14 (31.1%) | OR = 0.47 [0.19; 1.17] |
|  |  |  | GI | 20 (39.2%) |  | 21 (37.5%) | OR = 0.76 [0.32; 1.77] |
|  |  |  | G | 20 (40.0%) |  | 23 (43.4%) |  |
|  |  |  |  |  |  |  |  |
|  |  | Women  (n=306) | GA+ | 31 (57.4%) |  | 22 (37.3%) | OR = 0.75 [0.36; 1.57] |
|  |  |  | GA | 33 (40.2%) |  | 39 (45.9%) | OR = 1.32 [0.69; 2.51] |
|  |  |  | GI | 38 (41.8%) |  | 35 (36.5%) | OR = 0.95 [0.50; 1.79] |
|  |  |  | G | 43 (45.3%) |  | 42 (43.8%) |  |
|  |  |  |  |  |  |  |  |
| Sitting time | Health condition (n=266) | Men  (n=93) | GA+ | 625.18 (227.72) |  | 592.50 (274.41) | β = -31.44 [-133.93; 71.06] |
|  |  |  | GA | 630.00 (215.74) |  | 557.05 (234.41) | β = -59.39 [-159.69; 40.91] |
|  |  |  | GI | 490.79 (196.35) |  | 466.21 (204.42) | β = -41.18 [-129.43; 47.05] |
|  |  |  | G | 508.57 (190.47) |  | 525.19 (238.17) |  |
|  |  |  |  |  |  |  |  |
|  |  | Women  (n=173) | GA+ | 444.38 (190.52) |  | 477.86 (194.29) | β = 35.02 [-33.66; 103.70] |
|  |  |  | GA | 473.40 (184.04) |  | 445.20 (237.49) | β = -18.44 [-83.46; 46.57] |
|  |  |  | GI | 497.44 (200.88) |  | 468.21 (199.91) | β = -11.84 [-73.50; 49.81] |
|  |  |  | G | 491.61 (217.45) |  | 477.50 (204.30) |  |
|  | No health condition (n=142) | Men  (n=63) | GA+ | 530.77 (228.27) |  | 436.67 (165.08) | β = -68.56 [-179.07; 41.94] |
|  |  |  | GA | 547.73 (230.53) |  | 545.29 (215.35) | β = 21.27 [-72.72; 115.26] |
|  |  |  | GI | 564.47 (158.05) |  | 503.89 (192.60) | β = -29.91 [-126.27; 66.44] |
|  |  |  | G | 526.36 (202.78) |  | 505.89 (211.71) |  |
|  |  |  |  |  |  |  |  |
|  |  | Women  (n=79) | GA+ | 597.00 (188.92) |  | 633.33 (192.35) | β = **118.48** [25.58; 211.38]** |
|  |  |  | GA | 524.40 (150.28) |  | 554.35 (165.27) | β = **89.24** [20.10; 158.38]** |
|  |  |  | GI | 514.52 (173.41) |  | 503.12 (158.48) | β = 42.09 [-27.69; 111.87] |
|  |  |  | G | 480.68 (181.75) |  | 442.79 (152.59) |  |
|  |  |  |  |  |  |  |  |
| Perceived increase in physical activity | Health condition (n=337) |  | GA+ |  |  | 21 (33.3%) | OR = **2.40* [1.12; 5.15]** |
|  |  |  | GA |  |  | 24 (28.9%) | OR = 1.95 [0.94; 4.06] |
|  |  |  | GI |  |  | 20 (19.2%) | OR = 1.14 [0.55; 2.40] |
|  |  |  | G |  |  | 15 (17.2%) |  |
|  |  |  |  |  |  |  |  |
|  | No health condition (n=162) |  | GA+ |  |  | 13 (61.9%) | OR = **4.66** [1.62; 13.43]** |
|  |  |  | GA |  |  | 6 (13.6%) | OR = 0.45 [0.16; 1.28] |
|  |  |  | GI |  |  | 11 (28.2%) | OR = 1.13 [0.45; 2.80] |
|  |  |  | G |  |  | 15 (25.9%) |  |
|  |  |  |  |  |  |  |  |
|  |  |  |  | **Post-intervention measurement (T1)** | | **Follow-up measurement (T2) versus T1** | |
| Intention |  | Men  (n=203) | GA+ | 27 (73.0%) | OR = 0.74 [0.28; 1.94] |  |  |
|  |  |  | GA | 34 (69.4%) | OR = 0.62 [0.26; 1.49] |  |  |
|  |  |  | GI | 52 (85.2%) | OR = 1.58 [0.61; 4.09] |  |  |
|  |  |  | G | 44 (78.6%) |  |  |  |
|  |  |  |  |  |  |  |  |
|  |  | Women  (n=361) | GA+ | 60 (95.2%) | OR = **4.65* [1.32; 16.36]** |  |  |
|  |  |  | GA | 74 (83.1%) | OR = 1.15 [0.55; 2.40] |  |  |
|  |  |  | GI | 83 (80.6%) | OR = 0.97 [0.48; 1.92] |  |  |
|  |  |  | G | 86 (81.1%) |  |  |  |
|  |  |  |  |  |  |  |  |
| Intention strength |  | Men  T1 (n=203)  T2 (n=174) | GA+ | 5.27 (2.62) | β = -0.36 [-1.35; 0.64] | 5.78 (2.44) | β = 0.32 [-0.43; 1.08] |
|  |  |  | GA | 5.82 (2.40) | β = 0.19 [-0.73; 1.11] | 5.61 (2.31) | β = -0.25 [-0.95; 0.45] |
|  |  |  | GI | 6.34 (2.17) | β = 0.72 [-0.15; 1.59] | 5.66 (2.71) | β = -0.60 [-1.27; 0.06] |
|  |  |  | G | 5.63 (2.44) |  | 5.72 (2.33) |  |
|  |  |  |  |  |  |  |  |
|  |  | Women  T1 (n=361)  T2 (n=317) | GA+ | 6.94 (2.05) | β = **0.88** [0.23; 1.53]** | 6.45 (2.23) | β = 0.03 [-0.56; 0.62] |
|  |  |  | GA | 6.37 (2.12) | β = 0.31 [-0.28; 0.91] | 6.49 (2.25) | β = 0.34 [-0.19; 0.86] |
|  |  |  | GI | 6.66 (2.01) | β = **0.60* [0.04; 1.17]** | 6.92 (1.69) | β = **0.62* [0.11; 1.13]** |
|  |  |  | G | 6.06 (2.16) |  | 6.00 (1.94) |  |
|  |  |  |  |  |  |  |  |
| Commitment | Health condition  T1 (n=312)  T2 (n=273) | Men  T1 (n=97)  T2 (n=84) | GA+ | 6.65 (1.93) | β = -0.16 [-1.07; 0.75] | 5.29 (2.42) | β = -0.75 [-1.94; 0.44] |
|  |  |  | GA | 7.06 (0.87) | β = 0.23 [-0.66; 1.11] | 5.12 (2.17) | β = **-1.36* [-2.53; -0.20]** |
|  |  |  | GI | 7.34 (1.53) | β = 0.54 [-0.25; 1.34] | 5.68 (2.60) | β = **-1.07* [-2.12; -0.01]** |
|  |  |  | G | 6.81 (1.52) |  | 6.20 (1.99) |  |
|  |  |  |  |  |  |  |  |
|  |  | Women  T1 (n=215)  T2 (n=189) | GA+ | 7.39 (1.47) | β = **0.73* [0.13; 1.33]** | 6.82 (1.59) | β = 0.68 [-0.05; 1.41] |
|  |  |  | GA | 7.00 (1.46) | β = 0.32 [-0.24; 0.89] | 6.47 (2.18) | β = 0.51 [-0.18; 1.19] |
|  |  |  | GI | 7.48 (1.45) | β = **0.79** [0.26; 1.33]** | 7.06 (1.67) | β = **0.90** [0.24; 1.56]** |
|  |  |  | G | 6.67 (1.57) |  | 5.80 (1.61) |  |
|  |  |  |  |  |  |  |  |
|  | No health condition  T1 (n=148)  T2 (n=128) | Men  T1 (n=60)  T2 (n=50) | GA+ | 7.40 (1.26) | β = 0.24 [-0.75; 1.23] | 6.10 (2.51) | β = 0.91 [-1.25; 3.08] |
|  |  |  | GA | 7.50 (1.15) | β = 0.32 [-0.52; 1.16] | 5.84 (2.43) | β = 0.61 [-1.23; 2.46] |
|  |  |  | GI | 7.00 (1.00) | β = -0.18 [-1.04; 0.68] | 5.53 (2.67) | β = 0.70 [-1.18; 2.58] |
|  |  |  | G | 7.18 (1.47) |  | 5.00 (2.64) |  |
|  |  |  |  |  |  |  |  |
|  |  | Women  T1 (n=88)  T2 (n=78) | GA+ | 7.55 (1.37) | β = 0.66 [-0.35; 1.68] | 6.18 (2.68) | β = -0.42 [-1.92; 1.08] |
|  |  |  | GA | 7.04 (1.08) | β = 0.13 [-0.64; 0.89] | 6.76 (1.76) | β = 0.53 [-0.59; 1.65] |
|  |  |  | GI | 6.43 (1.63) | β = -0.48 [-1.26; 0.29] | 6.45 (2.16) | β = 0.61 [-0.53; 1.75] |
|  |  |  | G | 6.91 (1.49) |  | 6.16 (2.19) |  |
|  |  |  |  |  |  |  |  |
| Self-efficacy (composite score) | Health condition (n=312) |  | GA+ | 5.65 (1.63) | β = 0.32 [-0.25; 0.90] |  |  |
|  |  |  | GA | 5.70 (1.77) | β = 0.37 [-0.18; 0.93] |  |  |
|  |  |  | GI | 5.97 (1.79) | β = **0.64* [0.13; 1.16]** |  |  |
|  |  |  | G | 5.33 (1.75) |  |  |  |
|  |  |  |  |  |  |  |  |
|  | No health condition (n=148) |  | GA+ | 6.02 (1.58) | β = 0.29 [-0.60; 1.18] |  |  |
|  |  |  | GA | 6.00 (1.74) | β = 0.26 [-0.45; 0.97] |  |  |
|  |  |  | GI | 5.55 (1.75) | β = -0.19 [-0.91; 0.53] |  |  |
|  |  |  | G | 5.73 (1.67) |  |  |  |

Abbreviations: *IQR* interquartile range, *CI* confidence interval, *MET* metabolic equivalent of task, *SD* standard deviation, β regression coefficient, *OR* odds ratio
*P<.05
**P<.01

^a^ The median and (IQR) are reported as the distribution is skewed to the right
^b^ The results were log transformed for the analysis (using the natural logarithm) and subsequently back transformed

^c^ The ‘moderate/high’ category was compared to the ‘low’ category
