# Supplementary material for: Promoting an active choice among physically inactive adults: a randomised web-based four-arm experiment
Source: Int J Behav Nutr Phys Act. 2022 Apr 27;19:49. doi: 10.1186/s12966-022-01288-y (PMC9043878; doi:10.1186/s12966-022-01288-y)
Supplement: Supplementary file 3 — Additional file 3: Table S4. Participants' general quantitative evaluation of the interventionand questionnaire. Table S5. Qualitative analysis of participants' evaluation ofthe function and form of the intervention and questionnaire and participants’suggestions for improvement. Table S6. Participants’ quantitative evaluation of the extentto which the intervention supported deliberate decision making about physicalactivity. [file 12966_2022_1288_MOESM3_ESM.docx]

**Additional file 3: Process evaluation results**

**Landais et al. *Promoting an Active Choice among Physically Inactive Adults: A Randomised Web-Based Four-Arm Experiment***

**Table S4.** Participants’ general quantitative evaluation of the intervention and questionnaire

| **Item** | **Scale** | **Group** | **First measurement (T1)** | **Follow-up measurement (T2)** |
| --- | --- | --- | --- | --- |
|  |  |  | **Mean (SD)** | **Mean (SD)** |
|  |  |  |  |  |
| The information and exercises were well-organised. | 1 (strongly disagree) – 7 (strongly agree) | GA+ | 5.25 (1.44) |  |
|  |  | GA | 5.41 (1.28) |  |
|  |  | GI | 5.49 (1.15) |  |
|  |  | G | 5.31 (1.18) |  |
|  |  |  |  |  |
| The information and exercises were understandable to me. | 1 (strongly disagree) – 7 (strongly agree) | GA+ | 5.63 (1.19) |  |
|  |  | GA | 5.73 (1.07) |  |
|  |  | GI | 6.00 (0.96) |  |
|  |  | G | 5.67 (1.11) |  |
|  |  |  |  |  |
| The readability of the information and exercises was good. | 1 (strongly disagree) – 7 (strongly agree) | GA+ | 5.52 (1.28) |  |
|  |  | GA | 5.64 (1.21) |  |
|  |  | GI | 5.90 (0.96) |  |
|  |  | G | 5.51 (1.23) |  |
|  |  |  |  |  |
| The intervention was: | (1) not interesting - (5) interesting | GA+ | 3.86 (1.15) | 3.93 (1.17) |
|  |  | GA | 4.11 (1.07) | 3.98 (1.01) |
|  |  | GI | 4.12 (1.01) | 4.22 (0.90) |
|  |  | G | 3.86 (1.05) | 3.98 (0.93) |
|  |  |  |  |  |
| The intervention was: | (1) too short - (5) too long | GA+ | 3.30 (0.77) | 2.80 (0.51) |
|  |  | GA | 3.15 (0.68) | 2.73 (0.60) |
|  |  | GI | 2.90 (0.52) | 2.85 (0.46) |
|  |  | G | 3.03 (0.45) | 2.84 (0.42) |
|  |  |  |  |  |
| The intervention was: | (1) unclear - (5) clear | GA+ | 3.87 (1.10) | 4.31 (0.80) |
|  |  | GA | 4.13 (0.98) | 4.28 (0.80) |
|  |  | GI | 4.31 (0.85) | 4.37 (0.79) |
|  |  | G | 4.13 (1.06) | 4.36 (0.77) |
|  |  |  |  |  |
| The intervention was: | (1) unpleasant to complete - (5) pleasant to complete | GA+ | 3.70 (1.11) | 4.19 (0.91) |
|  |  | GA | 3.90 (1.08) | 4.03 (0.97) |
|  |  | GI | 4.07 (0.96) | 4.15 (0.92) |
|  |  | G | 3.93 (0.95) | 4.12 (0.86) |

Abbreviations: *SD* standard deviation

**Table S5.** Qualitative analysis of participants' evaluation of the function and form of the intervention and questionnaire and participants’ suggestions for improvement

| **Topic** | **Theme** | **Summary of answers to open-ended questions** | **Participants (n)** | **Quotes** |
| --- | --- | --- | --- | --- |
| **Function evaluation** | Intervention is clear, pleasant or motivates behaviour change | Some participants made a general positive comment about the intervention, for instance that it was clarifying or pleasant to complete, or that it motivated behaviour change. | 9 | "It was good and clear."  "It's good to write down what I already know deep down. It made me a bit emotional because I know that it's necessary for myself and for my health, but I seem to not consider myself important enough since I do not always act on it…" |
|  | Intervention increases awareness | A few participants reported that the intervention had helped them to become more aware about their physical activity behaviour and/or health in general. | 7 | “I've recently had an angioplasty, this really got me thinking."  “It was very clear and also clarified how important I consider something, and why I still don't make it important in my life." |
|  | Already aware and knowledgeable about physical activity | Some participants indicated that they were already aware of their physical activity behaviour or already knew the facts provided in the intervention. Most of these participants seemed to suggest that the intervention was not of added value and some participants explicitly stated this. Some participants explained that awareness and knowledge were not the ‘problem’ for their low levels of physical activity, but that certain barriers, including physical conditions, hindered them. A few participants indicated that they had already reflected on their physical activity behaviour and/or had already made a choice about it prior to the intervention. | 22 | “Of course I know that I should be more physically active! However, I think this questionnaire is not going to change that much, unfortunately."  "I've already made these considerations over the past years. I can't or I do not want to become more physically active because of constraints." |
|  | Intervention is not helpful/ topic is negatively evaluated | Some participants made a general negative comment about the intervention, including that it did not help or motivate. A few other participants made negative comments about the topic ‘physical activity’, for instance because they did not like to engage in physical activity. | 8 | "These exercises didn't help me. All questions already had an answer within the plan that we already have." |
|  | Unpleasant confrontation with own behaviour | Two participants commented that the intervention unpleasantly confronted them with their behaviour. | 2 | "Unpleasant questionnaire to complete because of confrontation with my own behaviour." |
|  | Paternalistic tone of intervention | Two participants negatively evaluated the tone of the intervention, commenting that it was too paternalistic, compelling or formal. | 2 | "The exercises made me feel a little mothered and very taken aback." |
| **Form evaluation** | Items are confusing, too long or very similar | Multiple participants indicated that some items were confusing, or that the wording of items was too complicated or too long. Three participants made comments about IPAQ items, including the wording and the way time was queried. Finally, two participants commented that items were very similar and one thought the items were too leading. | 12 | "Too long and complex questions; not inviting to complete." |
|  | Technical issues | Three participants wrote that they had trouble completing the questionnaire on their phone or tablet. One participant indicated that it was not possible to enter numbers and another participant thought it was annoying that it was necessary to tick a box before an answer could be entered. Furthermore, a visually impaired participant mentioned that the questionnaire was hard to read. | 6 | "Difficult to complete from a mobile phone/ small screen." |
| **Suggestions for improvement** | Take personal situation into account | Many participants indicated that physical impairments and disabilities, including fibromyalgia, multiple sclerosis, chronic obstructive pulmonary disease, rheumatism and a recent delivery, should be taken into account. In addition, some participants suggested to pay attention to individuals’ occupation or family situation (e.g. having young children). A few participants mentioned that the intervention should be more tailored in general. With regard to the questionnaire items, a few older participants indicated that age must be taken into account, as some have retired. In addition, the readability should be improved for visually impaired participants. Finally, two participants mentioned that COVID-19 restrictions must be taken into account. | 26 | "Take into account individuals with chronic illnesses where physical activity difficult or painful (e.g. rheumatism, multiple sclerosis, fibromyalgia, etc.)." |
|  | Include more detailed information and concrete examples | Multiple participants suggested to include more detailed information, especially about the specific health benefits of physical activity. Some participants suggested to include more concrete and detailed examples. Furthermore, a few participants proposed to focus more on the fun of physical activity, or to broaden the focus and to include eating behaviour as well. Finally, one participant suggested to make it less quantitative and another participant suggested to make it less ‘fat-phobic’ and to communicate ‘health at every size’. | 20 | "I'm missing the concrete health benefits of physical activity. For example: running 30 minutes equals burning X calories or reducing the cardiovascular disease risk by X percent. This could promote an even more deliberate choice." |
|  | Use visual examples and reduce the amount of text and questions | Participants’ suggestions about the lay-out concerned (a) using visual examples, such as images and videos; (b) using less text; (c) making in more clear, for instance by including blank lines; (d) using more similar response categories; and (e) shorting the questionnaire. | 10 | "Provide more detailed examples, something visual (e.g. video), something interactive." |
|  | Clarify and simplify wording of items | In accordance with participants’ previous comments that some items were confusing or too long, some participants suggested to clarify and simplify the wording of items. | 6 | “The questions are sometimes unnecessarily complicated and can easily be made more accessible." |
|  | Include advices to increase physical activity | Multiple participants suggested to include short advices in the intervention, for instance about how to integrate physical activity in one’s daily routine, how to be physically active with a physical impairment, how to cope with barriers or how to discuss physical activity possibilities with an employer. | 7 | “Provide advice to make physical activity easier. For example: Go grocery shopping by bike. This will make it more concrete." |
|  | Add certain items to the questionnaire | A few participants proposed to add a certain items to the questionnaire. One participant suggested to ask individuals what drives them to be physically active, as this could increase their motivation to become more active. Other participants suggested to add a question about why individuals do not engage in much physical activity, or about the levels of physical activity before the Covid-19 pandemic. | 5 | "To motivate individuals to become more physically active, you should know what drives them. For instance, do they engage in physical activity to prevent cardiovascular diseases, or to improve focus during work?" |
|  | Offer a pedometer | Two participants suggested to offer a pedometer with the intervention. One of them suggested to additionally include a coach, who would monitor participants’ steps. | 4 | "It sounds very childish, but maybe offer a physical activity application/pedometer at a reduced rate and a coach who coaches remotely. This coach should be able to see whether I'm physically active or not." |
|  | Improve display on small screens | In line with participants’ previous comments about the difficulties completing the questionnaire on their phone or tablet, one participant explicitly suggested to improve the display on mobile phones. | 1 | “Improve display on mobile phones” |

**Table S6.** Participants’ quantitative evaluation of the extent to which the intervention supported deliberate decision making about physical activity

| **Item** | **Scale** | **Group** |  | **First measurement (T1)** |
| --- | --- | --- | --- | --- |
|  |  |  |  | **Mean (SD)** |
|  |  |  |  |  |
| The information and exercises have made me more aware of my current physical activity behaviour | 1 (strongly disagree) – 7 (strongly agree) | GA+ |  | 4.64 (1.50) |
|  |  | GA |  | 4.45 (1.66) |
|  |  | GI |  | 4.33 (1.50) |
|  |  | G |  | 4.24 (1.49) |
|  |  |  |  |  |
| The information and exercises helped me to make a more deliberate choice about my physical activity behaviour. | 1 (strongly disagree) – 7 (strongly agree) | GA+ |  | 4.28 (1.46) |
|  |  | GA |  | 4.03 (1.57) |
|  |  | GI |  | 3.98 (1.45) |
|  |  | G |  | 3.99 (1.48) |
|  |  |  |  |  |
| To what extent did the exercises help you make a more deliberate choice about your physical activity behaviour? Please rate each exercise from 1 (very bad) to 10 (very good). |  |  |  |  |
|  |  |  |  |  |
| A. Writing down advantages and disadvantages of my current physical activity behaviour and of being more physically active^a^ | 1 (very bad) - 10 (very good) | GA+ |  | 5.98 (2.25) |
|  |  | GA |  | 5.93 (2.15) |
|  |  | GI |  | N/A |
|  |  | G |  | N/A |
|  |  |  |  |  |
| B. Indicating what I consider important, and how much time, effort and energy I spend on it^a^ | 1 (very bad) - 10 (very good) | GA+ |  | 6.16 (2.04) |
|  |  | GA |  | 6.17 (2.07) |
|  |  | GI |  | N/A |
|  |  | G |  | N/A |
|  |  |  |  |  |
| C. Writing down what kind of physical activity I want to do, how often, where and when^b^ | 1 (very bad) - 10 (very good) | GA+ |  | 5.95 (2.21) |
|  |  | GA |  | N/A |
|  |  | GI |  | N/A |
|  |  | G |  | N/A |
|  |  |  |  |  |
| D. Writing down barriers to physical activity^a^ | 1 (very bad) - 10 (very good) | GA+ |  | 6.24 (2.00) |
|  |  | GA |  | 6.07 (2.09) |
|  |  | GI |  | N/A |
|  |  | G |  | N/A |
|  |  |  |  |  |
| E. Plan how to cope with those barriers^b^ | 1 (very bad) - 10 (very good) | GA+ |  | 5.99 (1.95) |
|  |  | GA |  | N/A |
|  |  | GI |  | N/A |
|  |  | G |  | N/A |

Abbreviations: *SD* standard deviation, *N/A* not applicable

^a^ Exercise was only included in groups GA+ and GA ^b^ Exercise was only included in group GA+
